# Resource Use in Swedish Nursing Homes: A Repeated Cross‐Sectional Follow‐Up Study

**DOI:** 10.1002/gps.70228

**Published:** 2026-06-06

**Authors:** Liza Privosnik, Rebecca Baxter, Laura Corneliusson, Hugo Lövheim, Anders Sköldunger

**Affiliations:** ^1^ Department of Nursing Umeå University Umeå Sweden; ^2^ Department of Community Medicine and Rehabilitation Umeå University Umeå Sweden; ^3^ Department of Nursing Department of Neurobiology Care Sciences and Society Karolinska Institutet Umeå University Umeå Sweden

**Keywords:** health resources, neuropsychiatric symptoms, nursing home, older adults, resource use, Sweden

## Abstract

**Objectives:**

Nursing homes in Sweden provide housing and care for people aged 65 years or older who require assistance with everyday activities. An increasing number of nursing home residents have cognitive and functional decline, which can result in additional time needed for care provision. This study aimed to explore changes in resource use and associated factors in Swedish nursing homes over a 5‐year period.

**Methods:**

This repeated cross‐sectional study analyzed baseline (2013–2014) and follow‐up (2018–2019) proxy‐rated data from 4599 participants from the Swedish National Inventory of Care and Health in Residential Aged Care study. Resource use was measured using the Resource Use in Dementia scale. Descriptive statistics, *t*‐tests, chi‐square tests, and multiple linear regressions were performed.

**Results:**

Total resource use increased from 7.15 h/day to 7.83 h/day between baseline and follow‐up. The number of residents living in a dementia unit increased from 34.6% to 43%. Higher independence in activities of daily living was associated with lower total resource use at follow‐up while living in a dementia unit was associated with higher total resource use. Higher total resource use was associated with seven neuropsychiatric symptoms. For residents living in a dementia unit, four neuropsychiatric symptoms were associated with higher total resource use.

**Conclusions:**

Resource use in Swedish nursing homes increased between baseline and follow‐up. These results may inform future policy, financing, and implementation decisions to support resource utilization in nursing homes.

## Key Points

- Resource use in Swedish nursing homes increased between baseline and follow‐up.
- Living in a dementia unit and some neuropsychiatric symptoms were associated with higher resource use.
- Increased knowledge on resource use may inform future policies for Swedish nursing homes at the regional and national levels.

## 1 Introduction

Nursing homes (NH) in the Swedish context provide housing and care for people 65 years or older who require assistance with everyday activities and care when a needs assessment indicates that care at home is not possible [1]. Dementia units in Sweden are defined as wards where person‐centered care (PCC) places the person in the center, and care must be based on individual needs [2]. They are needs tested, regulated by Swedish law, and financed, in most cases, by the public sector from municipality taxes [3]. NH staffing in Sweden varies across municipalities, with their main responsibility to ensure NH residents live as well as possible, despite potential physical or cognitive decline [4]. One study estimated that 66.6% of Swedish NH residents had cognitive decline [5] which requires more time and resources [6, 7].

Resource use can be described as time spent doing personal activities of daily living (PADL), instrumental ADL (IADL), and supervision [8, 9, 10]. A study from Sweden found that NH residents with increased levels of cognitive impairment used more care time for PADL, IADL, and supervision [7, 8]. A recent overview of international resource utilization groups found that anxiety, decline in functional status, and aberrant motor behavior increased the care time residents received [11].

Increasing care time underscores the necessity to consider the number of available beds in Swedish NHs, which has declined over the last 10 years [12]. Staff in Swedish NHs report a lack of time as a barrier to supporting NH residents' needs and completing their other work tasks [13]. Lack of time and organizational issues can lead to missed care activities in NHs, which has consequences for NH residents' safety, quality of care, and overall satisfaction [14]. A Swedish study found that PCC in NHs improves NH residents' quality of life and reduces staff job strain without increasing resource use, which benefits both NH residents and staff [7].

The need for resources and care time to support residents' needs has risen due to the aging population [12]. To our knowledge, there have been no studies exploring changes in resource use over time among NH residents in Sweden. Therefore, this study aimed to explore changes in resource use and associated factors in Swedish NHs over a 5‐year period.

## 2 Materials and Methods

This study used a repeated cross‐sectional design. The data were collected at two time points as part of the Swedish National Inventory of Care and Health in Residential Aged Care study (SWENIS). The baseline data were collected between November 2013 and September 2014, and follow‐up data were collected between November 2018 and May 2019. The SWENIS study collected data on health, quality of life, and care organization in Swedish NHs [15].

### 2.1 Data Collection and Participants

Out of 290 municipalities in Sweden, 60 were randomly selected and invited to participate in the SWENIS I (S1) data collection. Forty‐seven municipal managers initially accepted an invitation to participate; however, five municipalities did not provide NH contact details, and five withdrew. A total of 202 NHs in 37 municipalities were contacted and provided with verbal and written information about the study. A total of 172 NHs from 35 municipalities participated in S1, resulting in 4831 completed proxy‐rated resident surveys [5]. Proxy‐raters were NH staff who knew the resident best and were used due to the expected high prevalence of cognitive impairment among NH residents, which would hinder self‐reported responses [8].

For the SWENIS II (S2) follow‐up data collection, a similar approach was used as in S1. The 35 municipalities that participated in S1 were invited to participate in S2, as well as 25 new municipalities. Forty‐nine municipal managers initially accepted the invitation; however, four municipalities withdrew, and two dropped out. A total of 3894 completed proxy‐rated resident surveys from 187 NHs in 43 municipalities were collected in S2 [16]. Surveys in both data collections were anonymized and coded for unit identification. The term ‘units’ refers to units within the NHs, and only the matched units of NHs in S1 and S2 were used in this study (*N* = 4599).

### 2.2 Instruments

#### 2.2.1 Resource Utilization in Dementia (RUD)

RUD was used to assess resource utilization in dementia concerning direct care activities. RUD reports time in hours and minutes over the day, utilizing a retrospective time frame from the previous week. RUD consists of three domains: personal activities of daily living (RUD PADL), instrumental activities of daily living (RUD IADL), and Supervision (RUD Supervision) [10, 17]. The RUD total represents the sum of minutes, hours, and days expressed in hours/day for each RUD domain. A higher number of hours/day indicates more resource utilization. The RUD scale has demonstrated good validity and reliability to measure resource use among community‐dwelling persons with dementia [9].

#### 2.2.2 Gottfries’ Cognitive Scale (GCS)

GCS was used to evaluate cognitive function. GCS comprises 27 statements assessing personal, environmental, and abstract orientation [18]. Each statement can be responded to with ‘yes’ (1) or ‘no’ (0), with a score below 24 indicating cognitive impairment. GCS has been previously validated for assessing the cognitive function of NH residents using staff proxy raters and has been recommended for survey‐based investigations in NH environments [19].

#### 2.2.3 The Katz Index of Independence in Activities of Daily Living (Katz ADL)

The Katz ADL was used to measure functional status in six categories: bathing, dressing, toileting, feeding, transferring, and continence [20]. Items are scored 1 or 0 and scores can range from 0 to 6, where a lower score corresponds to dependence in ADL [21]. Dependence is indicated when more than three activities indicate functional dependence [22]. The Katz ADL scale has been found to be a reliable and valid measurement of ADL among older people [23, 24].

#### 2.2.4 The Neuropsychiatric Inventory—Nursing Home Version (NPI‐NH)

NPI‐ NH was used to assess behavioral and psychological symptoms across 12 domains: delusions, hallucinations, agitation, depression, anxiety, euphoria, apathy, disinhibition, irritability, abnormal motor behavior, sleep/night disturbances, and appetite/eating changes [25]. Frequency is rated on a 4‐point Likert‐type scale ranging from 0 (never) to 4 (very often), and severity is rated on a 3‐point Likert‐type scale ranging from 1 (mild) to 3 (severe). The maximum possible score for each subscale is 12 (generated by multiplying frequency by severity), and the total score ranges from 0 to 144 (sum of 12 domains). A higher total score indicates higher frequency and severity of neuropsychiatric symptoms (NPS) [25, 26]. The NPI has been validated for use among persons with neuropsychiatric disorders [27].

### 2.3 Data Analysis

Descriptive statistics were used to describe and compare resource use among NH residents from matched units in S1 and S2. To explore differences between the S1 and S2, Chi‐squared tests and *T*‐tests were performed. To determine the normality and linearity of the data, visual inspection of the histograms and examining values for the skewness (± 2) and kurtosis (± 1) were performed [28].

Individual multiple linear regressions were utilized to explore how different factors were associated with resource use, with the dependent variables being resource use (total RUD, PADL, IADL, and Supervision) and the independent variables of age, sex, living in the dementia unit, NPI‐NH, Katz ADL, and GCS. Furthermore, to explore the association of the investigation year with resource use, multiple linear regression with interaction variables (living in the dementia unit, age, sex, NPI‐NH, Katz ADL, and GCS) was performed. Univariate linear regression was used to explore which NPS were associated with resource use (adjusted for age, sex, Katz ADL, living in dementia units, and GCS) as well as to explore which NPS were associated with resource use among residents living in dementia units (adjusted for age, sex, Katz ADL, living in dementia units, and GCS). In addition, multiple linear regression with interaction variables was used to explore which NPS drive resource use for residents living in dementia units.

Imputations for missing data for up to one item in NPI‐NH were replaced with the mean value of the individual for the total scale. For missing data for GCS, up to three missing items were imputed with 0.5 [19]. In RUD (all dimensions), imputations were performed on all variables marked as ‘missing’, ‘0 days’, and ‘8 days’ to ‘7’, given that the maximum days per week and score is 7. Cases with more than 168 h were marked as ‘missing’ across all dimensions, since the maximum number of hours per week is 168. Statistical significance was defined as *p* < 0.05 for the descriptive statistics, *t*‐tests, chi‐squared tests, multiple and univariate linear regressions to explore associations. For the multiple and univariate linear regressions exploring interactions, statistical significance was defined as *p* < 0.15. The analysis was performed using IBM SPSS Statistics, version 29.0.1.0. For Windows [29].

## 3 Results

The total number of NH residents who participated in S1 was 2,559, with a mean age of 85.68 years (Table 1). In S2, 2040 NH residents with a mean age of 85.54 years participated from the same NHs. Most residents in S1 and S2 were female, and no significant differences were found in residents' age, sex, cognitive function, ADL, and living with a partner. The proportion of people living in a dementia unit increased from 34.6% in S1 to 43.0% in S2 (*p* < 0.001). The mean total resource use increased from 7.12 in S1 to 7.83 in S2 (*p* = 0.004), and the mean NPI‐NH score decreased from 16.0 to 14.3 (*p* = 0.004).

**TABLE 1:** Characteristics of the study population.

	SWENIS I (*n* = 2559)		SWENIS II (*n* = 2040)			
	Mean (SD)	*n* (%)	Mean (SD)	*n* (%)	*p*‐value	Cohen's d
Age	85.68 (7.86)		85.54 (8.10)		0.562	
Sex						
Female		1731 (68.3)		1380 (68.1)	0.922	
Male		804 (31.7)		645 (31.9)		
Living in a dementia unit						
Yes		864 (34.6)		871 (43.0)	**< 0.001**	
No		1633 (65.4)		1154 (57.0)		
Living with a partner						
Yes		71 (2.8)		66 (3.3)	0.380	
No		2445 (97.2)		1952 (96.7)		
GCS score	17.16 (8.19)		16.88 (8.24)		0.243	0.034
Katz ADL	2.87 (2.11)		2.92 (2.13)		0.436	0.023
NPI‐NH	16.0 (19.9)		14.3 (17.2)		**0.004**	**0.091**
RUD total	7.12 (8.06)		7.83 (8.40)		**0.004**	**0.086**
PADL	1.85 (2.11)		1.98 (2.23)		0.051	0.059
IADL	1.34 (1.83)		1.47 (1.91)		**0.015**	**0.069**
Supervision	3.96 (6.50)		4.43 (6.98)		**0.019**	**0.069**

*Note:* Bold numbers indicate significant associations (*p* < 0.05).

Abbreviations: GCS, Gottfries’ Cognitive Scale; RUD, Resource Utilization in Dementia; RUD PADL, Activities of Daily Living; RUD IADL, Instrumental Activities of Daily Living; NPI‐NH, The Neuropsychiatric Inventory—Nursing Home version; Katz ADL, The Katz Index of Independence in Activities of Daily Living; Ref: reference.

The multiple linear regression showed that female sex was associated with higher PADL resource use (*p* = 0.028) in S1 (Table 2). In S2, female sex was associated with lower PADL resource use, but was not statistically significant. For total resource use, there was a differential effect (a significant interaction) of female sex between S1 and S2 (*p* = 0.084). Higher NPI scores were associated with higher total resource use (*p* < 0.001) in S1, but not in S2. For PADL resource use, there was a significant interaction between NPI‐NH score and investigation year (*p* = 0.031). Higher Katz ADL scores were associated with lower total resource use in S1 (*p* < 0.001) and S2 (*p* < 0.001), but the interaction was not significant. Higher GCS scores were associated with lower total resource use (*p* < 0.001) in S1, but not in S2. The interaction between GCS scores and the investigation year for total resource use indicated a significant differential effect (*p* = 0.007). Living in a dementia unit was associated with higher total resource use in S1 (*p* < 0.001) and S2 (*p* < 0.001), although the interaction was not significant. The standardized beta showed that lower GCS scores had the greatest effect on total resource use in S1, and living in a dementia unit had the greatest effect on total resource use in S2 (Supporting Information S1).

**TABLE 2:** Factors associated with resource use in S1 and S2, and association of the investigation year with resource use.

	RUD total			RUD PADL			RUD IADL			RUD supervision		
	S1	S2	*p*	S1	S2	*p*	S1	S2	*p*	S1	S2	*p*
	B(*p*)	B(*p*)		B(*p*)	B(*p*)		B(*p*)	B(*p*)		B(*p*)	B(*p*)	
Age	−0.022 (0.357)	0.006 (0.836)	0.093 (0.735)	−0.007 (0.285)	−0.005 (0.483)	−0.009 (0.896)	−0.007 (0.209)	−0.003 (0.560)	−0.010 (0.878)	−0.008 (0.669)	0.013 (0.570)	0.111 (0.633)
Sex (female)	0.308 (0.440)	−0.625 (0.176)	**−0.476 (0.084)**	**0.235 (0.028)**	−0.054 (0.636)	**−0.148 (0.038)**	0.154 (0.095)	−0.117 (0.253)	**−0.132 (0.034**)	0.106 (0.747)	−0.459 (0.248)	−0.187 (0.420)
NPI‐NH	**0.036 (< 0.001)**	0.023 (0.080)	−0.477 (0.101)	**0.007 (0.012)**	0.000 (0.959)	**−0.161 (0.031)**	0.004 (0.058)	0.005 (0.087)	−0.011 (0.872)	**0.026 (0.002)**	0.018 (0.105)	−0.298 (0.224)
Katz ADL	**−0.377 (< 0.001)**	**−0.612 (< 0.001)**	0.022 (0.938)	**−0.269 (< 0.001)**	**−0.315 (< 0.001)**	−0.001 (0.992)	**−0.063 (0.007)**	**−0.112 (< 0.001)**	−0.045 (0.469)	−0.051 (0.541)	−0.188 (0.059)	0.073 (0.754)
GCS	**−0.142 (< 0.001)**	−0.009 (0.805)	**0.755 (0.007)**	−0.013 (0.104)	0.010 (0.258)	**0.160 (0.028)**	−0.004 (0.600)	0.014 (0.070)	0.065 (0.311)	**−0.126 (< 0.001)**	−0.032 (0.286)	**0.534 (0.025)**
Living in a dementia unit	**2.067 (< 0.001)**	**3.080 (< 0.001)**	0.015 (0.958)	**0.235 (0.043)**	**0.340 (0.004)**	0.037 (0.606)	**0.370 (< 0.001)**	**0.532 (< 0.001)**	−0.024 (0.705)	**1.461 (< 0.001)**	**2.191 (< 0.001)**	−0.020 (0.932)

*Note:* Bold numbers in columns B(*p*) indicate significant associations (*p* < 0.05); bold numbers in columns *p* indicate significant associations (*p* < 0.15); *p* represents the interaction between time points and factor over 5 years.

Abbreviations: GCS, Gottfries’ Cognitive Scale; Ref: reference; NPI‐NH, The Neuropsychiatric Inventory—Nursing Home version; Katz ADL, The Katz Index of Independence in Activities of Daily Living; S1, SWENIS I; S2, SWENIS II; RUD, Resource Utilization in Dementia; RUD PADL, Activities of Daily Living; RUD IADL, Instrumental Activities of Daily Living.

The univariate linear regressions showed that seven NPI‐NH domains were associated with higher total resource use: Delusions (*p* = 0.030), Hallucinations (*p* = 0.009), Anxiety (*p* = 0.040), Disinhibition (*p* = 0.030), Irritability (*p* = 0.002), Aberrant Motor Behavior (*p* < 0.001), and Sleep/Night Behaviors (*p* < 0.001) (Table 3). Five NPI‐NH domains were associated with higher PADL resource use, four NPI‐NH domains were associated with higher IADL resource use, and four domains were associated with higher Supervision resource use.

**TABLE 3:** NPI‐ NH domains' association with resource use.

NPI‐NH Domain	RUD total B(*p*)	RUD PADL B(*p*)	RUD IADL B(*p*)	RUD supervision B(*p*)
Delusions	**0.116 (0.030)**	0.010 (0.471)	**0.030 (0.015)**	0.077 (0.088)
Hallucinations	**0.139 (0.009)**	**0.042 (0.002)**	**0.027 (0.026)**	0.074 (0.096)
Aggressions/Agitation	0.075 (0.165)	0.006 (0.662)	0.001 (0.962)	0.068 (0.133)
Depression/Dysphoria	0.088 (0.102)	006 (0.630)	0.010 (0.422)	0.073 (0.106)
Anxiety	**0.112 (0.040)**	0.007 (0.640)	0.014 (0.249)	0.92 (0.043)
Elation/Euphoria	0.043 (0.483)	−0.018 (0.262)	0.002 (0.872)	0.057 (0.266)
Apathy	0.086 (0.101)	**0.027 (0.045)**	0.014 (0.252)	0.044 (0.313)
Disinhibition	**0.123 (0.030)**	**0.038 (0.009)**	0.014 (0.272)	0.067 (0.158)
Irritability	**0.153 (0.002)**	0.012 (0.330)	0.001 (0.918)	**0.136 (0.001)**
Aberrant motor behavior	**0.306 (< 0.001)**	**0.032 (0.012)**	**0.032 (0.004)**	**0.242 (< 0.001)**
Sleep/Night behaviors	**0.170 (< 0.001)**	**0.035 (0.001)**	**0.045 (< 0.001)**	**0.092 (0.008)**
Appetite/Eating changes	0.087 (0.144)	0.012 (0.423)	0.014 (0.294)	0.062 (0.214)

*Note:* Adjusted for GCS, Katz ADL, Sex, Age, and living in a dementia unit; bold numbers indicate significant associations (*p* < 0.05), analysis performed on combined S1 and S2 matched‐unit data.

Abbreviations: GCS, Gottfries’ Cognitive Scale; Katz ADL, The Katz Index of Independence in Activities of Daily Living; Ref: reference; RUD, Resource Utilization in Dementia; RUD PADL, Activities of Daily Living; RUD IADL, Instrumental Activities of Daily Living.

The univariate linear regression showed that four NPI‐NH domains were associated with higher total resource use when living in a dementia unit: Apathy (*p* = 0.042), Irritability (*p* = 0.031), Aberrant Motor Behavior (*p* = < 0.001), Sleep/Night Behaviors (*p* = 0.040) (Table 4). Four NPI‐NH domains were associated with higher PADL resource use, and three NPI‐NH domains were associated with higher Supervision resource use. One NPI‐NH domain was associated with lower PADL resource use. None of the NPI‐NH domains were associated with higher or lower IADL resource use. Four NPI‐NH domains were associated with higher total resource use when living in a general unit: Hallucination (*p* = 0.007), Disinhibition (*p* = 0.008), Aberrant Motor Behavior (*p* = < 0.001), and Sleep/Night Behaviors (*p* = < 0.001). Three NPI‐NH domains were associated with higher PADL resource use, seven NPH‐NI domains were associated with higher IADL resource use, and two NPI‐NH domains were associated with higher Supervision resource use.

**TABLE 4:** NPI‐NH domains' association with the resource use of NH residents living in a dementia unit, and interaction between NPI‐NH domains' association with resource use and living in dementia units.

NPI‐NH domain	RUD total			RUD PADL			RUD IADL			RUD supervision		
	Living in a dementia unit		*p*	Living in a dementia unit		*p*	Living in a dementia unit		*p*	Living in a dementia unit		*p*
	No B(*p*)	Yes B(*p*)		No B(*p*)	Yes B(*p*)		No B(*p*)	Yes B(*p*)		No B(*p*)	Yes B(*p*)	
Delusions	0.097 (0.135)	0.128 (0.160)	0.018 (0.892)	0.019 (0.429)	0.000 (0.999)	−0.031 (0.355)	**0.038 (0.010)**	0.020 (0.342)	−0.13 (0.663)	0.039 (0.474)	0.108 (0.160)	0.060 (0.587)
Hallucinations	**0.172 (0.007)**	0.092 (0.320)	−0.049 (0.707)	**0.059 (< 0.001)**	0.021 (0.345)	−0.035 (0.306)	**0.053 (< 0.001)**	−0.004 (0.844)	−**0.052 (0.077)**	0.069 (0.191)	0.074 (0.344)	0.022 (0.839)
Aggression/Agitation	0.083 (0.214)	0.060 (0.502)	−0.062 (0.629)	0.005 (0.801)	0.008 (0.702)	−0.010 (0.762)	0.026 (0.089)	−0.025 (0.242)	**−0.072 (0.013)**	0.052 (0.360)	0.077 (0.304)	0.018 (0.871)
Depression/Dysphoria	0.053 (0.410)	0.133 (0.157)	0.107 (0.426)	−0.023 (0.489)	−0.014 (0.530)	−0.043 (0.204)	0.018 (0.199)	0.000 (0.985)	−0.024 (0.425)	0.013 (0.813)	0.149 (0.059)	**0.171 (0.133)**
Anxiety	0.093 (0.178)	0.130 (0.143)	−0.036 (0.781)	0.032 (0.092)	−0.018 (0.397)	**−0.069 (0.039)**	**0.047 (0.002)**	−0.017 (0.407)	**0.077 (0.008)**	0.017 (0.770)	**0.165 (0.026)**	0.102 (0.347)
Elation/Euphoria	0.110 (0.152)	−0.034 (0.741)	**−0.216 (0.101)**	0.014 (0.499)	**−0.052 (0.033)**	**−0.074 (0.027)**	0.031 (0.071)	−0.028 (0.246)	**−0.048 (0.106)**	0.065 (0.312)	0.043 (0.623)	−0.102 (0.358)
Apathy	−0.004 (0.949)	**0.171 (0.042)**	0.066 (0.615)	−0.010 (0.600)	**0.061 (0.002)**	**0.062 (0.066)**	0.012 (0.415)	0.015 (0.438)	−0.014 (0.624)	−0.011 (0.843)	0.096 (0.174)	0.018 (0.869)
Disinhibition	**0.187 (0.008)**	0.048 (0.605)	−0.132 (0.299)	**0.049 (0.011)**	0.025 (0.256)	−0.012 (0.722)	**0.039 (0.014)**	−0.011 (0.629)	**−0.050 (0.082)**	0.093 (0.117)	0.033 (0.669)	−0.068 (0.526)
Irritability	0.120 (0.054)	**0.178 (0.031)**	−0.032 (0.810)	0.008 (0.649)	0.018 (0.367)	0.023 (0.514)	0.005 (0.718)	−0.003 (0.872)	−0.030 (0.319)	0.101 (0.054)	**0.165 (0.017)**	−0.018 (0.873)
Aberrant motor behavior	**0.316 (< 0.001)**	**0.298 (< 0.001)**	0.086 (0.507)	0.014 (0.660)	**0.049 (0.010)**	0.038 (0.247)	**0.040 (0.005)**	0.026 (0.160)	−0.013 (0.664)	**0.261 (< 0.001)**	**0.223 (< 0.001)**	0.057 (0.599)
Sleep/Night behaviors	**0.178 (< 0.001)**	**0.151 (0.040)**	0.048 (0.729)	**0.031 (0.025)**	**0.039 (0.028)**	0.029 (0.409)	**0.059 (< 0.001)**	0.027 (0.114)	−0.001 (0.965)	**0.091 (0.026)**	0.084 (0.170)	0.017 (0.885)
Appetite/Eating changes	0.134 (0.055)	0.014 (0.896)	−0.126 (0.348)	0.012 (0.533)	0.012 (0.606)	0.006 (0.855)	**0.036 (0.020)**	−0.013 (0.601)	**−0.064 (0.029)**	0.085 (0.150)	0.019 (0.831)	−0.065 (0.568)

*Note:* Bold numbers in columns B(*p*) indicate significant associations (*p* < 0.05); bold numbers in columns *p* indicate significant associations (*p* < 0.15); *p* represents the interaction between living in dementia units and NPS association with resource use, analysis made on combined S1 and S2 matched units data.

Abbreviations: GCS, Gottfries’ Cognitive Scale; Katz ADL, The Katz Index of Independence in Activities of Daily Living; Ref: reference; RUD, Resource Utilization in Dementia; RUD PADL, Activities of Daily Living; RUD IADL, Instrumental Activities of Daily Living.

Multiple linear regression showed a significant interaction between Elation/Euphoria and dementia unit versus general unit for total resource use (Table 4). Three significant interactions were seen for NPI‐NH domains concerning PADL resource use, six significant interactions for IADL resource use, and one significant interaction for Supervision resource use were seen, indicating that some neuropsychiatric symptoms were driving resource use differently in dementia units as compared to general units.

## 4 Discussion

This study aimed to explore the changes in resource use and associated factors in Swedish NH over 5 years. Total resource use increased between S1 and S2. The number of residents living in dementia units also increased, and living in a dementia unit was associated with higher resource use in both S1 and S2. This is in line with a recent report that estimated the prevalence of dementia and related resource use has been steadily rising [30]. Living in a dementia unit was a strong driver of total resource use in both investigation years, and given that this proportion increased significantly between baseline and follow‐up, it may explain why total resource use increased, even though the mean ADL and GCS scores for the residents did not change.

This study found that a high level of independence in ADL was associated with lower resource use in S1 and S2, which is consistent with earlier studies [6, 8, 31]. In both sex and GCS, there was a significant change in interaction over 5 years. Further, higher levels of cognitive function were associated with lower PADL resource use and were a more important driver of resource use in S1. This is in line with a previous study, which reported that lower cognitive function was associated with more total care time [32]. This could be due to more NH residents living in dementia units in S2, which may have reduced the workload of NH staff in general units.

The present study found that higher NPI scores were more important drivers of PADL resource use in S1 compared to S2. A previous study based on the same sample found that the prevalence of some NPS between S1 and S2 had decreased, specifically in anxiety and sleep/night behaviors [33]. While there are no concrete explanations for these changes over time, they could be cautiously linked to changes in the care system at the national level, including the implementation of PCC. The increasing adoption of PCC during this period [34, 35], which emphasizes individualized support and has been linked to greater staff engagement, care intensity, and closer relationships between staff and NH residents without increasing resource use [7, 36, 37]. These results could point to a shift toward more personalized and efficient NH care, also indicated by the increased proportion of individuals with dementia living in specialized dementia units. Despite the overall decrease in prevalence of NPS, this study showed that symptoms such as delusions, hallucinations, anxiety, disinhibition, irritability, apathy, abnormal motor behaviors, and sleep/night behaviors were associated with higher total resource use. This is in line with previous research suggesting that apathy and hallucinations contribute to increased care costs [38] and imply that a wide range of NPS have substantial demands on NH staff [39].

Among the NH residents living in dementia units, those with symptoms of Elation/Euphoria were associated with lower resource use. On the contrary, anxiety, apathy, irritability, aberrant motor behavior, and sleep/night behaviors were associated with higher resource use in dementia units. Symptoms such as apathy, delusions, and dysphoria are strongly linked to cognitive impairment in dementia [40], which could further increase resource utilization and care complexity. Nonpharmacological interventions, specifically individualized caregiver training, aim to improve care quality for older people with cognitive disorders/dementia [41, 42]. In a previous study, individualized caregiver training significantly reduced the impact of NPS [43]. In the present study, depression/dysphoria and elation/euphoria were more important drivers of resource use in dementia units compared to general units. An earlier study from England found that agitation was a key driver of costs in residents with dementia [44]. Another found that NH residents with dementia were rated as having more NPS; however, when living in dementia units, those symptoms were better managed [45]. The care practices in NHs have been said to influence NPS [46]. Likewise, the care setting and environment are known to influence how NPS are perceived, prioritized, and translated into care needs [47]. NH staff working in dementia units may rate NPS as less severe due to greater familiarity and training, which can affect both perception and documentation of NPS. In addition, frequent communication between NH staff and residents, as well as assessing and meeting NH residents' needs, has been shown to reduce NPS [46]. This may, to some extent, explain the relationship between NPS as drivers of resource use in dementia units. Further studies could look into whether dementia units are beneficial for NH residents.

### 4.1 Strengths and Limitations

The data were drawn from a repeated cross‐sectional design in Sweden and therefore may only be generalizable to similar contexts. While NH staff who knew the NH resident best were used as proxy raters, recall and observer bias are possible. Nevertheless, proxy raters are commonly used to study populations who may be unable to provide accurate self‐reports [19]. In NH settings, some activities are carried out simultaneously, which may result in overestimation or underestimation of care time. Assessed time for RUD supervision includes time spent in the NH's common areas, where more than one resident is usually present, which may influence due to joint production. Lastly, there is a risk of overestimation in RUD imputation for days, where ‘missing’ was imputed with ‘7’. The study is strengthened by a relatively large representative sample that can provide a broad representation of the Swedish NH population.

## 5 Conclusion

To the best of our knowledge, this is the first study exploring factors associated with resource use and changes over time in Swedish NHs over time. By identifying the factors that drive resource use and how it has changed over time, it might be possible for staff to focus on alleviating NPS, which could, in turn, result in reduced resource use overall. In contrast, cognitive and functional decline may be harder to influence. This might be achieved by staff education and non‐pharmacological interventions such as PCC. These findings could help NH managers, stakeholders, and municipalities in future policy‐making decisions, financing, and implementation of PCC at the regional or national levels. Further follow‐up studies are needed to explore trends and to explore other factors that influence resource use.

## Funding

This research project was funded by the Swedish Research Council [Vetenskapsrådet] (2014–02715) and the Swedish Research Council for Health, Working Life and Welfare [FORTE] (2014–04016). The funding body had no involvement in study design; in the collection, analysis, and interpretation of data; in the writing of the report; and in the decision to submit the article for publication.

## Ethics Statement

Ethical approval was granted by the Swedish Regional Ethical Review Board for SWENIS 1 (Dnr: 2013/269‐31) and for SWENIS 2 from the Central Ethical Review Board (Dnr: 2018/145‐31).

## Conflicts of Interest

The authors declare no conflicts of interest.

## Supporting information


Supporting Information S1

## Data Availability

The data that support the findings of this study are available on request from the corresponding author. The data are not publicly available due to privacy or ethical restrictions.
