# Supplementary material for: Resource Use in Swedish Nursing Homes: A Repeated Cross‐Sectional Follow‐Up Study
Source: Int J Geriatr Psychiatry. 2026 Jun 6;41(6):e70228. doi: 10.1002/gps.70228 (PMC13242244; doi:10.1002/gps.70228)
Supplement: Supplementary file 1 — Supporting Information S1 [file GPS-41-e70228-s001.docx]

Supplementary File

Standardized betas for Table 2

|  | RUD total | | RUD PADL | | RUD IADL | | RUD Supervision | |
| --- | --- | --- | --- | --- | --- | --- | --- | --- |
|  | S1 | S2 | S1 | S2 | S1 | S2 | S1 | S2 |
|  | b | b | b | b | b | b | b | b |
| Age | -0.022 | 0.006 | -0.025 | -0.018 | -0.031 | -0.016 | -0.010 | 0.015 |
| Sex (Female) | 0.018 | -0.036 | 0.052 | -0.012 | 0.041 | -0.031 | -0.008 | -0.031 |
| NPI-NH | 0.091 | 0.048 | 0.061 | 0.001 | 0.048 | 0.048 | 0.078 | 0.045 |
| Katz ADL | -0.100 | -0.160 | -0.267 | -0.331 | -0.075 | -0.135 | -0.017 | -0.058 |
| GCS | -0.147 | -0.008 | -0.049 | 0.038 | -0.017 | 0.064 | -0.160 | -0.037 |
| Living in a dementia unit | 0.124 | 0.187 | -0.053 | -0.083 | -0.101 | -0.149 | 0.108 | 0.158 |
